# Mitigation of Aerosols Generated During Rhinologic Surgery: A Pandemic-Era Cadaveric Simulation

**DOI:** 10.1177/0194599820951169

**Published:** 2020-08-11

**Authors:** Dhruv Sharma, Michael J. Ye, Vincent J. Campiti, Kolin E. Rubel, Thomas S. Higgins, Arthur W. Wu, Taha Z. Shipchandler, Michael W. Sim, Sarah J. Burgin, Elisa A. Illing, Jae Hong Park, Jonathan Y. Ting

**Affiliations:** 1 Department of Otolaryngology–Head and Neck Surgery, Indiana University, Indianapolis, Indiana, USA; 2 School of Medicine, Indiana University, Indianapolis, Indiana, USA; 3 Department of Otolaryngology–Head and Neck Surgery, University of Louisville, Louisville, Kentucky, USA; 4 Rhinology, Sinus, and Skull Base, Kentuckiana Ear, Nose, and Throat, Louisville, Kentucky, USA; 5 Department of Otolaryngology–Head and Neck Surgery, Cedars Sinai, Los Angeles, California, USA; 6 School of Health Sciences, Purdue University, West Lafayette, Indiana, USA

**Keywords:** sinus surgery, skull base surgery, airborne, aerosol-generating procedure, endonasal drilling, SARS-CoV-2, COVID-19, aerosol particles

## Abstract

**Objective:**

After significant restrictions initially due to the COVID-19 pandemic, otolaryngologists have begun resuming normal clinical practice. However, the risk of SARS-CoV-2 transmission to health care workers through aerosolization and airborne transmission during rhinologic surgery remains incompletely characterized. The objective of this study was to quantify the number concentrations of aerosols generated during rhinologic surgery with and without interventions involving 3 passive suction devices.

**Study Design:**

Cadaver simulation.

**Setting:**

Dedicated surgical laboratory.

**Subjects and Methods:**

In a simulation of rhinologic procedures with and without different passive suction interventions, the concentrations of generated aerosols in the particle size range of 0.30 to 10.0 µm were quantified with an optical particle sizer.

**Results:**

Functional endoscopic sinus surgery with and without microdebrider, high-speed powered drilling, use of an ultrasonic aspirator, and electrocautery all produced statistically significant increases in concentrations of aerosols of various sizes (*P* < .05). Powered drilling, ultrasonic aspirator, and electrocautery generated the highest concentration of aerosols, predominantly submicroparticles <1 µm. All interventions with a suction device were effective in reducing aerosols, though the surgical smoke evacuation system was the most effective passive suction method in 2 of the 5 surgical conditions with statistical significance (*P* < .05).

**Conclusion:**

Significant aerosol concentrations were produced in the range of 0.30 to 10.0 µm during all rhinologic procedures in this cadaver simulation. Rhinologic surgery with a passive suction device results in significant mitigation of generated aerosols.

The severe acute respiratory syndrome coronavirus 2 (SARS-CoV-2) is responsible for the ongoing coronavirus disease 2019 (COVID-19) pandemic. In an effort to mitigate the spread of SARS-CoV-2, otolaryngologists across the United States have curtailed the majority of their clinical practice for months^1^ in compliance with Centers for Disease Control and Prevention recommendations.^2^ As the rate of new cases has stabilized and the strain on the hospital system and personal protective equipment (PPE) stores has reduced, the otolaryngology community has begun returning to clinical practice. However, concern remains over how to safely resume practice due to the significant lack of information regarding the risk of viral transmission associated with otolaryngologic procedures in the operating room and in the clinic.

Endonasal procedures have garnered significant attention due to the high viral load in the nasal cavity and nasopharynx.^3,4^ The existing literature suggests that with good technique, endonasal surgery with powered instruments such as drills can be performed with minimal production of droplets,^5^ which are thought to be the main mode of viral transmission of SARS-CoV-2.^6,7^ Despite these findings, there remains a potential for airborne aerosolization of viral particles within particles generated during rhinologic procedures.^8-11^ Therefore, the quantification of aerosols is critical in determining when a procedure can be performed safely and what level of PPE is required. To guide these safe practices, this study was designed to quantify the concentration of generated aerosols during various rhinologic procedures with a real-time particle-measuring instrument and to determine the effectiveness of available passive suction devices in reducing aerosols.

## Materials and Methods

### Supplies and Equipment

This study was exempt from the Indiana University School of Medicine institutional review board because it involved the use of nonliving deidentified human cadaveric tissue specimens (protocol 2004100753). The experiments in this study were all conducted in a dedicated surgical laboratory on a fresh-frozen cadaver head specimen at room temperature. The surgical laboratory was equipped with a HEPA filtration system (high-efficiency particulate air), which was used in between experimental conditions until aerosol levels returned to baseline.

### Experimental Setup and Aerosol Sampling

**Figure 1.**
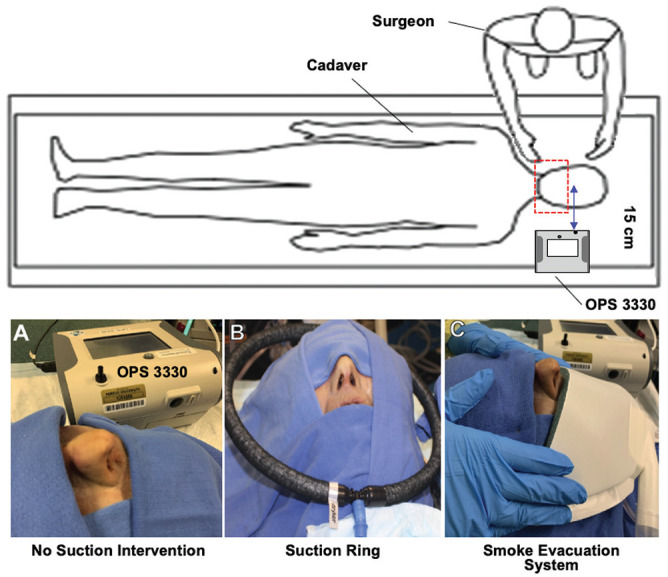
Experimental setup. (A) The optical particle sizer was positioned 15 cm from the midline columella. (B) The suction ring was manually held at the level of the nasal tip. (C) Placement of the smoke evacuation system.

The cadaver head was placed in the standard rhinologic position for an operating room procedure. All procedures were performed by a fellowship-trained right-handed rhinologist (J.Y.T.). Sampling of aerosols was performed with an optical particle sizer (OPS 3330; TSI Inc), which measures particle number concentration by size from 0.30 to 10.0 µm (16 channels per decade). The sampling flow rate through the 3-mm inlet port of the OPS 3330 was 1.0 L/min and calibrated with a flow calibrator (DryCal DC-Lite; BIOS) before and after sampling. **Table 1** shows the 16 particle diameter size ranges, measured in micrometers. Total number concentration of aerosols within each size range was recorded, and the size ranges of generated particles were measured every second during the experiments. The inlet port of the OPS 3330 was positioned 15 cm from the midline columella across from the surgeon (**Figure 1A**).

**Table 1.** Number Concentration of Generated Aerosols Above Baseline During Rhinologic Procedures.

	Number concentration of generated aerosols, particles/cm^3^				
Particle size, µm	Cold FESS	Microdebrider FESS	Powered drilling	Needle tip electrocautery	Ultrasonic aspirator
0.30-0.37	**1.18** ^b^	**−0.27** ^c^	**7.74** ^b^	**1.18** ^b^	**3.01** ^b^
0.37-0.47	8.44 × 10^−2^	7.47 × 10^−2^	**2.35** ^b^	**0.22** ^b^	**0.87** ^b^
0.47-0.58	2.89 × 10^−2^	3.40 × 10^−2^	**0.78** ^b^	**0.11** ^c^	**0.34** ^b^
0.58-0.72	1.07 × 10^−5^	1.46 × 10^−2^	**0.23** ^b^	2.48 × 10^−2^	**0.13** ^c^
0.72-0.90	1.86 × 10^−2^	2.60 × 10^−3^	**0.13** ^b^	2.40 × 10^−2^	**0.10** ^c^
0.90-1.12	1.24 × 10^−2^	**1.60 × 10** ^−2c^	**7.23 × 10** ^−2b^	2.58 × 10^−2^	1.73 × 10^−2^
1.12-1.39	2.46 × 10^−6^	**1.34 × 10** ^−2b^	**3.01 × 10** ^−2b^	7.20 × 10^−2^	8.12 × 10^−3^
1.39-1.73	−3.80 × 10^−3^	−5.00 × 10^−3^	**4.47 × 10** ^−2b^	−2.09 × 10^−2^	9.18 × 10^−3^
1.73-2.16	−8.81 × 10^−3^	**2.60 × 10** ^−2b^	**2.91 × 10** ^−2b^	2.46 × 10^−2^	−1.19 × 10^−2^
2.16-2.69	−3.00 × 10^−3^	**1.18 × 10** ^−2c^	**1.81 × 10** ^−2b^	−2.70 × 10^−3^	**−2.55 × 10** ^−2c^
2.69-3.34	−8.01 × 10^−3^	**1.78 × 10** ^−2b^	6.04 × 10^−3^	1.55 × 10^−3^	−1.55 × 10^−2^
3.34-4.16	−3.20 × 10^−3^	6.81 × 10^−3^	6.53 × 10^−3^	1.83 × 10^−4^	−1.40 × 10^−2^
4.16-5.18	**−7.01 × 10** ^−3c^	**1.44 × 10** ^−2b^	1.02 × 10^−3^	−3.64 × 10^−3^	**−1.05 × 10** ^−2c^
5.18-6.45	−4.00 × 10^−4^	4.20 × 10^−3^	3.52 × 10^−3^	−5.28 × 10^−3^	−6.01 × 10^−3^
6.45-8.03	1.00 × 10^−3^	**6.81 × 10** ^−3c^	2.01 × 10^−3^	−5.92 × 10^−3^	4.12 × 10^−6^
8.03-10.0	−3.20 × 10^−3^	4.40 × 10^−3^	−9.99 × 10^−4^	−2.46 × 10^−3^	1.34 × 10^−6^
Total	**1.29** ^b^	−2.52 × 10^−2^	**11.4** ^b^	**1.58** ^b^	**4.41** ^b^

Abbreviation: FESS, functional endoscopic sinus surgery.

a Bold indicates significance.

b *P* < .01.

c *P* < .05.

### Experiment

The HEPA filtration system was run for at least 3 minutes, followed by intranasal suctioning to evacuate any retained particulates in the surgical field after each experimental condition. Prior to each experiment, background aerosol concentration was measured every second for 1 minute. The following surgical procedures were performed systematically for 5 minutes each:

- Left functional endoscopic sinus surgery (FESS) with cold nonpowered instrumentation
- Left FESS with cold nonpowered instrumentation with a suction ring around the cadaver head (suction ring with tubing; connected to Neptune 2 [Stryker] on a maximum 520 mm Hg of suction; **Figure 1B**)
- Left FESS with cold nonpowered instrumentation with surgical smoke evaluation system (miniSQUAIR, Nascent Surgical; connected to Neptune 2 on high vacuum with 100% power; **Figure 1C**)
- Right FESS with cold powered suction microdebrider (Entellus Medical Shaver System SS-100, Stryker; connected to Neptune 2 on maximum 520 mm Hg) at 5000 rpm
- Right microdebrider FESS with suction ring
- Right microdebrider FESS with surgical smoke evaluation system.

Next, the following surgical procedures were performed for 2 minutes each:

- High-speed powered drilling (Pi Drive Motor, 5407-100-000; Stryker) of the sphenoid bone with a 4-mm cutter burr at 75,000 rpm
- Powered drilling of the sphenoid bone with rigid suction (Frazier suction, size 10) in the ipsilateral anterior nasal cavity
- Powered drilling of the sphenoid with rigid suction and suction ring
- Powered drilling of the sphenoid bone with rigid suction and surgical smoke evacuation system
- Use of an ultrasonic aspirator on frontal bone (model UST-2001, Ultrasonic Surgical System [Stryker]; 100% power, 50% suction, 15-mL/min irrigation)
- Use of an ultrasonic aspirator on frontal bone with rigid suction
- Use of an ultrasonic aspirator on frontal bone with rigid suction and suction ring
- Use of an ultrasonic aspirator on frontal bone with rigid suction and surgical smoke evaluation system
- Needle tip electrocautery
- Needle tip electrocautery with rigid suction
- Needle tip electrocautery with rigid suction and suction ring
- Needle tip electrocautery with rigid suction and surgical smoke evaluation system.

### Statistical Analysis

All statistical analyses were performed with SPSS Statistics for Windows version 20.0 (IBM Corp). Two-tailed *t* tests assuming unequal variance were used to compare the concentrations of aerosols generated from each experimental condition with the baseline concentrations prior to each experimental condition. A 1-way analysis of variance (ANOVA) was performed to compare the aerosol concentrations during the various experimental conditions for each procedure. Two-tailed *t* tests with Bonferroni correction for multiple comparisons were then used for post hoc testing. Due to the heterogeneity of variances, an independent samples Kruskal-Wallis *H* test was performed to compare the total aerosol concentrations between procedures with post hoc Mann-Whitney *U* tests with Bonferroni correction for multiple comparisons. Statistical significance was determined at *P* < .05.

## Results

### Comparison of Aerosol Generation Among Procedures

**Figure 2.**
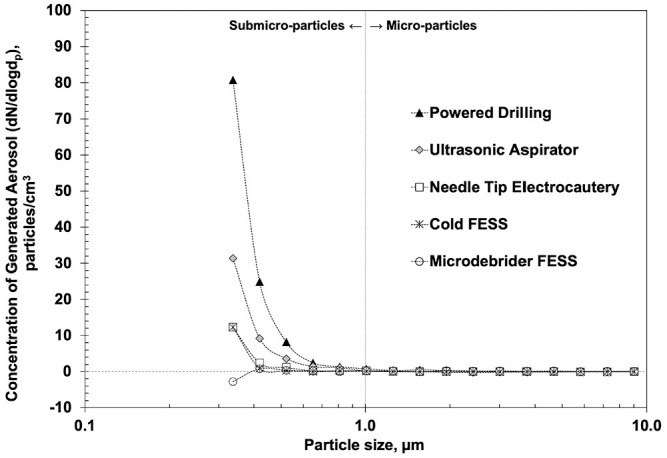
Mean concentration of generated aerosols over baseline levels for common rhinologic procedures. FESS, functional endoscopic sinus surgery.

A Kruskal-Wallis *H* test demonstrated a statistically significant difference in total aerosol concentrations generated among cold FESS, microdebrider FESS, powered drilling, needle tip electrocautery, and use of an ultrasonic aspirator, *H*^2^(4) = 476.191 (*P* < .001). Powered drilling produced a mean total aerosol concentration of 11.4 particles/cm^3^, which was significantly higher than cold FESS (vs 1.29 particles/cm^3^, *U* = 225.887, *P* < .001), microdebrider FESS (vs –0.025 particles/cm^3^, *U* = 503.350, *P* < .001), and needle tip electrocautery (vs 1.58 particles/cm^3^, *U* = 179.944, *P* < .001). There was no significant difference between powered drilling and the ultrasonic aspirator (vs 4.41 particles/cm^3^, *U* = −14.283, *P* > .99). The ultrasonic aspirator produced the second-highest total aerosol concentration, which was significantly greater than cold FESS (*U* = 240.170, *P* < .001), microdebrider FESS (*U* = 517.633, *P* < .001), and needle tip electrocautery (*U* = 194.227, *P* < .001). Needle tip electrocautery had a higher total aerosol concentration than microdebrider FESS (*U* = 323.406, *P* < .001), but the concentration was not significantly different from cold FESS (*U* = 45.943, *P* > .999). Last, microdebrider FESS had the lowest total aerosol concentration, which was significantly lower than cold FESS (*U* = 277.463, *P* < .001). **Figure 2** shows the concentration of generated aerosols from each rhinologic procedure. The y-axis is the concentration of generated aerosols, particles/cm^3^, on a logarithmic scale, which is the difference of aerosols from the experimental condition subtracted from the baseline levels. The x-axis is particle size diameter in micrometer. Submicron or submicroparticle aerosols are defined by a size range of 0.30 to 1.00 µm and microparticle aerosols by 1.00 to 10.0 µm. **Table 1** shows the mean concentration of generated aerosols stratified according to particle size.

### Aerosol Generation and Mitigation During FESS

**Figure 3.**
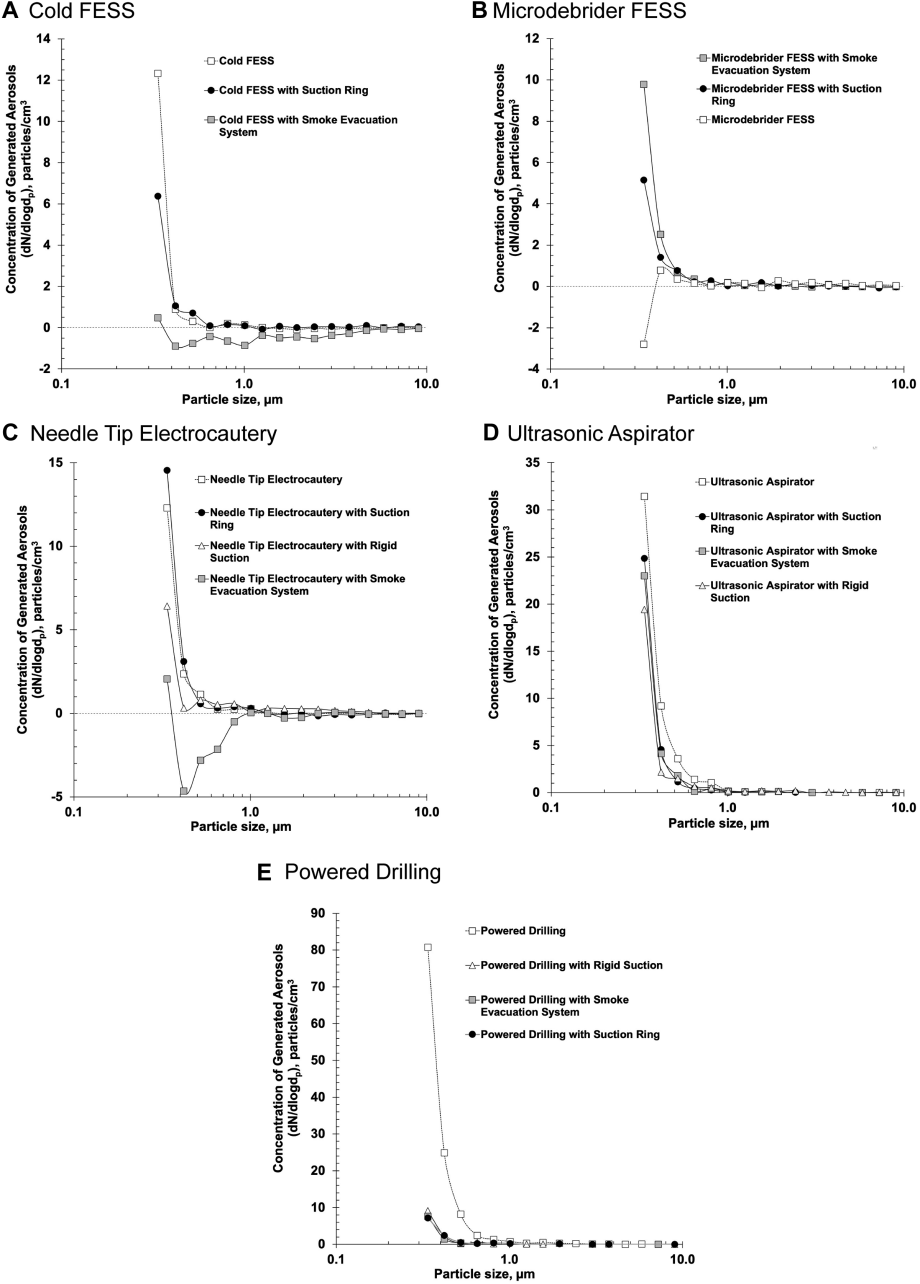
Mean concentration of generated aerosols over baseline with and without passive suction interventions: (A) cold FESS, (B) microdebrider FESS, (C) powered drilling, (D) needle tip electrocautery, (E) ultrasonic aspirator. FESS, functional endoscopic sinus surgery.

FESS with nonpowered instrumentation (cold FESS) generated a statistically significant increase in total aerosols (mean difference, 1.29 particles/cm^3^; *P* < .001), and there was a significant increase in the size range of 0.30 to 0.37 µm (*P* < .001). One-way ANOVA comparing aerosol concentrations generated during cold FESS without suction, with suction ring, and with the surgical smoke evacuation system demonstrated a significant difference among conditions for total concentration and all particle size ranges (*P* < .001 for each). **Table 2** shows the maximum total number concentration of generated aerosols for each experimental condition. Use of the surgical smoke evacuation system resulted in significantly decreased aerosol concentrations (*P* < .001) in each particle size range, except 8.03 to 10.0 µm as compared with FESS with no suction (*P* = .27) and 5.18 to 6.45 µm as compared with FESS with the ring suction (*P* = .07; **Figure 3A**, **Table 3**).

**Table 2.** Maximum Total Number Concentrations of Generated Aerosols During Rhinologic Procedures With and Without Suction Interventions.

	Maximum total number concentration of generated aerosols, particles/cm^3^				
Condition	Cold FESS	Microdebrider FESS	Sphenoid drilling	Needle tip electrocautery	Ultrasonic aspirator
Alone	42.9	32.0	954.6	55.1	536.6
Rigid suction	—	—	34.8	39.3	66.7
Suction ring	35.5	31.4	36.6^a^	62.6^a^	63.9^a^
SSES	18.7	48.4	37.1^a^	21.6^a^	54.5^a^

Abbreviation: SSES, surgical smoke evacuation system.

a Condition also included rigid suction.

**Table 3.** One-Way ANOVA Post Hoc Analyses of Aerosol Generation for Cold FESS Conditions.

Particle size range, µm	Condition 1	Condition 2	Mean difference,^a^ particles/cm^3^	*P* value
0.30-0.37	Alone^b^	Ring^c^	0.63	<.001
		SSES	1.25	<.001
	Ring		0.62	<.001
0.37-0.47	Alone	SSES	0.15	<.001
	Ring		0.19	<.001
0.47-0.58	Alone	Ring	−3.62 × 10^−2^	.010
		SSES	0.11	<.001
	Ring		0.14	<.001
0.58-0.72	Alone	SSES	4.31 × 10^−2^	<.001
	Ring		5.02 × 10^−2^	<.001
0.72-0.90	Alone	SSES	8.58 × 10^−2^	<.001
	Ring		7.54 × 10^−2^	<.001
0.90-1.12	Alone	SSES	9.86 × 10^−2^	<.001
	Ring		9.22 × 10^−2^	<.001
1.12-1.39	Alone	SSES	3.97 × 10^−2^	<.001
	Ring		3.59 × 10^−2^	<.001
1.39-1.73	Alone	SSES	4.63 × 10^−2^	<.001
	Ring		5.51 × 10^−2^	<.001
1.73-2.16	Alone	SSES	4.07 × 10^−2^	<.001
	Ring		4.37 × 10^−2^	<.001
2.16-2.69	Alone	SSES	4.71 × 10^−2^	<.001
	Ring		5.17 × 10^−2^	<.001
2.69-3.34	Alone	SSES	2.99 × 10^−2^	<.001
	Ring		3.47 × 10^−2^	<.001
3.34-4.16	Alone	SSES	2.39 × 10^−2^	<.001
	Ring		2.55 × 10^−2^	<.001
4.16-5.18	Alone	Ring	−1.06 × 10^−2^	<.001
		SSES	9.42 × 10^−3^	<.001
	Ring		2.00 × 10^−2^	<.001
5.18-6.45	Alone	Ring	3.60 × 10^−3^	.032
		SSES	6.82 × 10^−3^	<.001
6.45-8.03	Alone	SSES	1.02 × 10^−2^	<.001
	Ring		1.12 × 10^−2^	<.001
8.03-10.00	Alone	Ring	−4.80 × 10^−3^	<.001
	Ring	SSES	6.61 × 10^−3^	<.001
Total	Alone	Ring	0.53	<.001
		SSES	1.99	<.001
	Ring		1.46	<.001

Abbreviations: ANOVA, analysis of variance; FESS, functional endoscopic sinus surgery; SSES, surgical smoke evacuation system.

a Condition 1 – condition 2.

b *Alone* indicates FESS with nonpowered instrumentation.

c *Ring* indicates suction ring.

There was no significant difference in total particle concentration during FESS performed with powered suction microdebrider as compared with baseline (mean difference, –0.025 particles/cm^3^; *P* = .83). However, a significant decrease in aerosol concentration was noted in the particle size range of 0.30 to 0.37 µm (*P* = .021), and significant increases in aerosol concentrations were detected in 7 particle size ranges (**Table 1**). Comparing this condition with the ring suction and the surgical smoke evacuation system by 1-way ANOVA revealed significant differences for total concentration and all particle size ranges (*P* < .05 for each). Both suction interventions resulted in decreased aerosol concentrations at larger particle sizes but increased concentrations at smaller particle sizes as compared with the suctioning microdebrider alone (**Figure 3B**, **Table 4**).

**Table 4.** One-Way ANOVA Post Hoc Analyses of Aerosol Generation for Microdebrider FESS Conditions.

Particle size range, µm	Condition 1	Condition 2	Mean difference,^a^ particles/cm^3^	*P* value
0.30-0.37	Alone^b^	Ring^c^	−0.76	<.001
		SSES	−1.20	<.001
	Ring		−0.44	<.001
0.37-0.47	Alone	Ring	−5.81 × 10^−2^	.047
		SSES	−0.16	<.001
	Ring		−0.16	<.001
0.47-0.58	Alone	Ring	−3.89 × 10^−2^	.008
		SSES	−3.33 × 10^−2^	.03
0.58-0.72	Alone	SSES	−1.86 × 10^−2^	.041
0.72-0.90	Alone	Ring	−2.38 × 10^−2^	.001
	Ring	SSES	2.12 × 10^−2^	.003
0.90-1.12	Ring	SSES	−1.50 × 10^−2^	.035
1.12-1.39	Alone	SSES	9.81 × 10^−3^	.023
1.39-1.73	Alone	Ring	−2.22 × 10^−2^	<.001
		SSES	−1.22 × 10^−2^	.017
	Ring		1.00 × 10^−2^	.07
1.73-2.16	Alone	Ring	2.40 × 10^−2^	<.001
		SSES	2.20 × 10^−2^	<.001
2.16-2.69	Alone	SSES	1.00 × 10^−2^	.012
2.69-3.34	Alone	Ring	1.42 × 10^−2^	<.001
		SSES	2.00 × 10^−2^	<.001
3.34-4.16	Ring	SSES	−7.81 × 10^−3^	.02
4.16-5.18	Alone	Ring	1.26 × 10^−2^	<.001
		SSES	1.38 × 10^−2^	<.001
5.18-6.45	Alone	SSES	5.80 × 10^−3^	.007
6.45-8.03	Alone	Ring	1.40 × 10^−2^	<.001
		SSES	4.60 × 10^−3^	.042
	Ring		−9.41 × 10^−3^	<.001
8.03-10.00	Alone	Ring	6.81 × 10^−3^	<.001
		SSES	5.60 × 10^−3^	<.001
Total	Alone	Ring	−0.81	<.001
		SSES	−1.35	<.001
	Ring		−0.54	<.001

Abbreviations: ANOVA, analysis of variance; FESS, functional endoscopic sinus surgery; SSES, surgical smoke evacuation system.

a Condition 1 – condition 2.

b *Alone* indicates FESS with powered microdebrider.

c *Ring* indicates suction ring.

### Aerosol Generation and Mitigation During Endonasal Powered Drilling

High-speed endonasal powered drilling of the sphenoid rostrum generated a significant increase in total aerosol concentration as compared with baseline (mean difference, 11.44 particles/cm^3^; *P* < .001) with significant increases of particles ranging from 0.30 to 2.69 µm (**Table 1**). One-way ANOVA comparing aerosol generation from drilling without suction, with rigid suction, with rigid suction and suction ring, and with rigid suction plus the surgical smoke evacuation system showed significant differences for total concentration and the first 12 of 16 particle size ranges, up to 4.16 µm (0.30-3.34 µm, *P* < .001 for each; 3.34-4.16 µm, *P* = .04). All 3 suction intervention conditions had significantly decreased aerosol concentrations as compared with no suction for the particles ranging in size of 0.30 to 2.69 µm; rigid suction plus the surgical smoke evacuation system also had decreased concentrations at larger particle sizes (*P* < .001; **Figure 3C**, **Table 5**). Rigid suction plus the surgical smoke evacuation system resulted in significantly decreased aerosol concentrations as compared with rigid suction alone in 3 particle size ranges.

**Table 5.** One-Way ANOVA Post Hoc Analyses of Aerosol Generation for Powered Drilling Conditions.

Particle size range, µm	Condition 1	Condition 2	Mean difference,^a^ particles/cm^3^	*P* value
0.30-0.37	Alone^b^	Rigid^c^	6.86	<.001
		Rigid + ring^d^	7.05	<.001
		Rigid + SSES	7.01	<.001
0.37-0.47	Alone	Rigid	2.14	<.001
		Rigid + ring	2.12	<.001
		Rigid + SSES	2.21	<.001
0.47-0.58	Alone	Rigid	0.76	<.001
		Rigid + ring	0.73	<.001
		Rigid + SSES	0.74	<.001
0.58-0.72	Alone	Rigid	0.18	<.001
		Rigid + ring	0.21	<.001
		Rigid + SSES	0.26	<.001
	Rigid		8.11 × 10^−2^	.017
0.72-0.90	Alone	Rigid	0.11	<.001
		Rigid + ring	9.10 × 10^−2^	<.001
		Rigid + SSES	0.13	<.001
	Rigid + ring		4.25 × 10^−2^	.022
0.90-1.12	Alone	Rigid	8.78 × 10^−2^	<.001
		Rigid + ring	5.13 × 10^−2^	<.001
		Rigid + SSES	9.43 × 10^−2^	<.001
	Rigid	Rigid + ring	−3.65 × 10^−2^	.003
	Rigid + ring	Rigid + SSES	4.30 × 10^−2^	<.001
1.12-1.39	Alone	Rigid	2.71 × 10^−2^	<.001
		Rigid + ring	3.26 × 10^−2^	<.001
		Rigid + SSES	3.91 × 10^−2^	<.001
1.39-1.73	Alone	Rigid	3.71 × 10^−2^	<.001
		Rigid + ring	4.87 × 10^−2^	<.001
		Rigid + SSES	6.97 × 10^−2^	<.001
	Rigid		3.25 × 10^−2^	<.001
	Rigid + ring		2.10 × 10^−2^	.049
1.73-2.16	Alone	Rigid	3.81 × 10^−2^	<.001
		Rigid + ring	1.81 × 10^−2^	.025
		Rigid + SSES	3.86 × 10^−2^	<.001
	Rigid	Rigid + ring	−2.00 × 10^−2^	.009
	Rigid + ring	Rigid + SSES	2.05 × 10^−2^	.007
2.16-2.69	Alone	Rigid	2.41 × 10^−2^	<.001
		Rigid + ring	3.26 × 10^−2^	<.001
		Rigid + SSES	3.46 × 10^−2^	<.001
2.69-3.34	Alone	Rigid + SSES	1.76 × 10^−2^	<.001
	Rigid		1.65 × 10^−2^	<.001
	Rigid + ring		1.35 × 10^−2^	.005
Total	Alone	Rigid	10.3	<.001
		Rigid + ring	10.4	<.001
		Rigid + SSES	10.7	<.001

Abbreviations: ANOVA, analysis of variance; SSES, surgical smoke evacuation system.

a Condition 1 – condition 2.

b *Alone* indicates endonasal powered drilling without suction.

c *Rigid* indicates rigid suction device.

d *Ring* indicates suction ring.

### Aerosol Generation and Mitigation With Endonasal Needle Tip Electrocautery

Needle tip electrocautery of the nasal mucosa along the septum and inferior turbinate without suction demonstrated a significant increase in total aerosol concentration as compared with baseline (mean difference, 1.58 particles/cm^3^; *P* < .001) with significant increases in 3 particle size ranges (0.30-0.58 µm; **Table 1**). In comparing this condition with the aerosol concentrations generated during each of the 3 interventional conditions, 1-way ANOVA showed a significant difference for 14 of the 16 particle size ranges (0.30-0.90 µm and 1.12-8.03 µm: *P* = .003). Rigid suction plus the surgical smoke evacuation system resulted in the greatest decrease in aerosol generation, with concentrations significantly lower than rigid suction alone in 10 particle size ranges (*P* = .015; **Figure 3D**, **Table 6**).

**Table 6.** One-Way ANOVA Post Hoc Analyses of Aerosol Generation for Needle Tip Electrocautery Conditions.

Particle size range, µm	Condition 1	Condition 2	Mean difference,^a^ particles/cm^3^	*P* value
0.30-0.37	Alone^b^	Rigid^c^	0.56	<.001
		Rigid + SSES	0.98	<.001
	Rigid	Rigid + ring^d^	−0.78	<.001
		Rigid + SSES	0.42	.016
	Rigid + ring		1.20	<.001
0.37-0.47	Alone	Rigid	0.19	.014
		Rigid + SSES	0.66	<.001
	Rigid	Rigid + ring	−0.26	<.001
		Rigid + SSES	0.47	<.001
	Rigid + ring		0.73	<.001
0.47–0.58	Alone	Rigid + SSES	0.38	<.001
	Rigid		0.35	<.001
	Rigid + ring		0.32	<.001
0.58–0.72	Alone	Rigid + SSES	0.23	<.001
	Rigid		0.25	<.001
	Rigid + ring		0.23	<.001
0.72-0.90	Alone	Rigid + SSES	7.19 × 10^−2^	<.001
	Rigid		0.10	<.001
	Rigid + ring		8.57 × 10^−2^	<.001
0.90–1.12	Alone	Rigid	−2.20 × 10^−2^	.046
	Rigid	Rigid + ring	2.96 × 10^−2^	.002
		Rigid + SSES	2.91 × 10^−2^	.003
1.12-1.39	Alone	Rigid	−2.92 × 10^−2^	.009
	Rigid	Rigid + ring	3.26 × 10^−2^	.002
		Rigid + SSES	5.29 × 10^−2^	<.001
1.39-1.73	Alone	Rigid	−2.36 × 10^−2^	.002
		Rigid + SSES	2.62 × 10^−2^	<.001
	Rigid	Rigid + ring	2.42 × 10^−2^	.001
		Rigid + SSES	4.97 × 10^−2^	<.001
	Rigid + ring		2.55 × 10^−2^	.001
1.73-2.16	Alone	Rigid	−2.52 × 10^−2^	<.001
	Rigid	Rigid + ring	3.91 × 10^−2^	<.001
		Rigid + SSES	2.46 × 10^−2^	<.001
	Rigid + ring		−1.45 × 10^−2^	.041
2.16-2.69	Alone	Rigid	−1.42 × 10^−2^	.018
	Rigid	Rigid + ring	2.27 × 10^−2^	<.001
	Rigid + ring	Rigid + SSES	−1.34 × 10^−2^	.031
3.34-4.16	Rigid	Rigid + ring	2.06 × 10^−2^	<.001
	Rigid + ring	Rigid + SSES	−1.47 × 10^−2^	.003
4.16-5.18	Alone	Rigid	−1.00 × 10^−2^	.015
	Rigid	Rigid + ring	1.30 × 10^−2^	.001
		Rigid + SSES	1.00 × 10^−2^	.015
5.18-6.45	Alone	Rigid	−6.92 × 10^−3^	.020
		Rigid + ring	−7.74 × 10^−3^	.006
6.45-8.03	Alone	Rigid	−7.38 × 10^−3^	.001
	Rigid	Rigid + ring	7.19 × 10^−3^	.001
Total	Alone	Rigid	0.59	.003
		Rigid + SSES	2.38	<.001
	Rigid	Rigid + ring	−0.81	<.001
		Rigid + SSES	1.79	<.001
	Rigid + ring		2.60	<.001

Abbreviations: ANOVA, analysis of variance; SSES, surgical smoke evacuation system.

a Condition 1 – condition 2.

b *Alone* indicates endonasal needle tip electrocautery without suction.

c *Rigid* indicates rigid suction device.

d *Ring* indicates suction ring.

### Aerosol Generation and Mitigation With Use of an Ultrasonic Aspirator

The use of an ultrasonic aspirator on frontal bone resulted in significant increases in total aerosol concentration (mean difference, 4.41 particles/cm^3^; *P* < .001) and 5 particle size ranges <1 µm (**Table 1**). In comparing this and the aerosol concentrations associated with each of the 3 interventional conditions, 1-way ANOVA showed a significant difference between conditions for total concentration and 11 of the 16 particle size ranges: 0.30 to 0.72 µm and 1.73 to 8.03 µm (*P* = .045). All 3 suction interventions resulted in significantly decreased aerosol concentrations at smaller particle sizes but significantly increased concentrations at larger sizes (**Figure 3E**, **Table 7**). Conditions from both the rigid suction plus suction ring and the rigid suction plus surgical smoke evacuation system had significantly decreased aerosol concentrations as compared with the rigid suction alone at multiple particle size ranges.

**Table 7.** One-Way ANOVA Post Hoc Analyses of Aerosol Generation for Ultrasonic Aspirator Conditions.

Particle size range, µm	Condition 1	Condition 2	Mean difference,^a^ particles/cm^3^	*P* value
0.30-0.37	Alone^b^	Rigid^c^	1.15	<.001
		Rigid + ring^d^	0.63	.043
		Rigid + SSES	0.81	.003
0.37-0.47	Alone	Rigid	0.67	<.001
		Rigid + ring	0.44	<.001
		Rigid + SSES	0.48	<.001
0.47-0.58	Alone	Rigid	0.20	.016
		Rigid + ring	0.23	.003
0.58-0.72	Alone	Rigid + SSES	0.12	.039
1.73-2.16	Alone	Rigid	−2.50 × 10^−2^	.038
2.16-2.69	Alone	Rigid	−4.45 × 10^−2^	<.001
		Rigid + ring	−2.85 × 10^−2^	<.001
		Rigid + SSES	−1.80 × 10^−2^	.012
	Rigid	Rigid + ring	1.60 × 10^−2^	.035
		Rigid + SSES	2.65 × 10^−2^	<.001
2.69-3.34	Alone	Rigid + SSES	−1.65 × 10^−2^	.001
3.34-4.16	Alone	Rigid	−2.05 × 10^−2^	<.001
		Rigid + ring	−1.00 × 10^−2^	.008
		Rigid + SSES	−9.01 × 10^−3^	.023
	Rigid	Rigid + ring	1.05 × 10^−2^	.004
		Rigid + SSES	1.15 × 10^−2^	.001
4.16-5.18	Alone	Rigid	−1.30 × 10^−2^	<.001
5.18-6.45	Alone	Rigid	−6.01 × 10^−3^	.021
		Rigid + SSES	−6.01 × 10^−3^	.021
6.45-8.03	Rigid	Rigid + ring	6.51 × 10^−3^	.040
		Rigid + SSES	1.00 × 10^−2^	<.001
Total	Alone	Rigid	2.00	<.001
		Rigid + ring	1.44	.019
		Rigid + SSES	1.58	.007

Abbreviations: ANOVA, analysis of variance; SSES, surgical smoke evacuation system.

a Condition 1 – condition 2.

b *Alone* indicates endonasal ultrasonic aspiration without suction.

c *Rigid* indicates rigid suction device.

d *Ring* indicates suction ring.

## Discussion

As the return to clinical practice begins, many otolaryngologists remain wary of performing endoscopic endonasal procedures given the high viral loads found in the upper respiratory specimens of patients^3^ with COVID-19 and the potential risk of aerosolization and airborne transmission of SARS-CoV-2. Therefore, it is critical to not only quantify the concentration and particle size ranges of aerosols generated from different rhinologic procedures but also study the mitigating effects of available suction devices in reducing aerosols.

There has been some recent confusion in the literature regarding the concept and definition of aerosols, so we believe that it is important to clarify the terminology.^12^ Aerosols are defined as particles suspended in a gas that can contain a variety of pathogens, including viruses, and those particles with a diameter <5.0 to 10.0 µm are known to be capable of short- and long-range transmission as well as penetration into the lower airway.^10^ Airborne transmission of aerosols generally refers to transmission by particles <10.0 µm, which is defined by the Infectious Diseases Society of America as “respirable particles.”^10,13^ SARS-CoV-2 virions are measured to be 60 to 140 nm and as a result can be transported via aerosols,^14^ and there are a number of cases reported that can be explained only by aerosol-based transmission.^8^ However, the scientific community continues to debate how to classify aerosols and acknowledges that strict size criteria can be arbitrary and should be carefully interpreted according to the environmental condition.^10,15^ In addition, the risk of viral transmission posed by aerosols has not been quantified, and it remains unknown what particle concentrations and duration of exposure to classify as dangerous.

With this context in mind, the findings from our cadaveric surgical simulation indicate that all of the studied rhinologic procedures—including both types of FESS (nonpowered instrumentation and microdebrider), powered drilling, use of an ultrasonic aspirator, and needle tip electrocautery—generated a statistically significant increase in the number concentration of aerosols from 0.30 to 10.0 µm, predominantly in the submicroparticle range from 0.30 to 1.0 µm. This is a novel finding within the otolaryngology literature, as the previously published article on aerosol generation from endonasal procedures reported findings in the microparticle range of 1.0 to 10.0 µm.^16^

In this study, the quantity of generated aerosols varied significantly among procedures, with powered drilling producing the greatest concentration and microdebrider FESS producing the least. The finding of high-speed endonasal powered drilling carrying the greatest risk of generating aerosols is consistent with that of Workman et al^16^; however, our study found that the majority of aerosols generated by the drill were submicroparticles (<1.0 µm). This simulation also differs in that we report aerosol production during FESS with and without a powered suction microdebrider. The majority of aerosols generated during cold FESS were 0.30 to 0.37 µm. In comparison, microdebrider FESS had much lower overall aerosol concentration for each particle size, likely secondary to active suctioning. Otherwise, our results showed significant aerosol generation from needle tip electrocautery, though we found that an ultrasonic aspirator generated even more aerosols. It is important to note that both studies used the same machine, though there was a difference in its positioning. In this simulation, the OPS 3330 was placed across from the surgeon, on the right side of the cadaver head, in an effort to more accurately represent the aerosol risk to the operating surgeon and surgical technologist. Workman et al also positioned the machine 15 cm away from the nares, though they placed it directly inferior to the nares.

This simulation tested 3 passive suction interventions that all significantly reduced aerosols in multiple size ranges for all the tested surgical conditions. Among these, the surgical smoke evacuation system appeared to be the most effective in mitigating aerosol generation, though a statistically significant difference was observed during cold FESS and electrocautery. As shown in **Figure 3B**, the concentration of generated aerosols during the microdebrider FESS conditions varied significantly in the range of 0.30 to 0.37 µm, and this variation may have been secondary to the microdebrider functioning as an active powered suction device.

Several limitations to this cadaveric simulation merit discussion. First and foremost, we measured the concentration of generated aerosols in the range from 0.30 to 10.0 µm and did not study the presence of viral particles or their infectious ability, aerodynamic properties, desiccation, and deposition patterns. Although our experiment does show that different suction interventions can reduce aerosol concentrations generated during rhinologic surgery, we cannot say whether this translates into reduction of infectious transmission risk or make recommendations on the use of PPE, as the presence of bacterial or viral pathogens in the aerosol was not assessed. Furthermore, aerosols were measured at a single fixed point, across from the surgeon. Therefore, the generated aerosols measured during the various procedures may reflect exposure to only the surgeon and surgical technologist. Further studies should measure aerosol levels at the average distance of anesthesia and circulating staff. Moreover, aerosol generation during surgery on live patients may be different than a cadaver head for several potential reasons: normal physiologic temperature and blood flow, intranasal secretions, or disease conditions such as nasal polyposis. Therefore, future studies measuring aerosol production during rhinologic surgery on patients in the operating room are necessary, and we recommend that these studies include the measurement of particles in the size range of 0.30 to 10.0 µm.

## Conclusion

Significant aerosol concentrations were produced in the range of 0.30 to 10.0 µm during all rhinologic procedures in this cadaver simulation, with high-speed powered endonasal drilling generating the greatest aerosol levels and microdebrider FESS producing the least. The majority of aerosols were produced in the submicroparticle range of <1.0 µm. Performing rhinologic procedures with a passive suction device is recommended to mitigate aerosol generation during surgery.
